# The Role of Neuroaxis Irradiation in the Treatment of Intraspinal Ewing Sarcoma: A Review and Meta-Analysis

**DOI:** 10.3390/cancers14051209

**Published:** 2022-02-25

**Authors:** Fabian M. Troschel, Kai Kröger, Jan J. Siats, Kambiz Rahbar, Hans Theodor Eich, Sergiu Scobioala

**Affiliations:** 1 Department of Radiation Oncology, Münster University Hospital, 48149 Münster, Germany; kai.kroeger@ukmuenster.de (K.K.); jan.siats@ukmuenster.de (J.J.S.); hans.eich@ukmuenster.de (H.T.E.); 2 Department of Nuclear Medicine, Münster University Hospital, 48149 Münster, Germany; kambiz.rahbar@ukmuenster.de

**Keywords:** intraspinal Ewing sarcoma, craniospinal irradiation, radiotherapy, overall survival, multimodal therapy

## Abstract

**Simple Summary:**

Primary extraosseous intraspinal Ewing sarcoma (EwS) is a rare disease and optimal treatment strategies remain unclear. Most patients undergo trimodal therapy consisting of surgery, chemotherapy and radiation therapy. In this study, we focus on the role of radiation therapy in the treatment of EwS, specifically the use of craniospinal irradiation (CSI). We identified 24 patients with intraspinal EwS treated with CSI. A majority of these patients achieved complete remission. We found that patients with multiple lesions at time of diagnosis and intradural tumor location were more likely to undergo CSI while patients with a single lesion received focal irradiation more often. In spite of this imbalance, there was no difference in survival outcome between treatment groups. In summary, CSI is a valuable option in the treatment of EwS and should be considered individually based on tumor and patient characteristics.

**Abstract:**

The role of cranio-spinal irradiation (CSI) for primary extraosseous intraspinal Ewing sarcoma (EwS) remains unclear. Here, we evaluate clinical and survival outcomes in patients with primary intraspinal EwS treated with CSI as part of multimodal primary therapy regimens. We abstracted patient information, including details on treatment application, efficacy, and tolerance from the literature and our hospital database for a cohort of 24 primary intraspinal EwS patients treated with CSI. Median age was 25.5 years, median CSI dose was 36 Gy and mean boost dose was 12.8 Gy. Sixteen patients (66.7%) achieved complete radiological remission, another 5 patients demonstrated partial response and 1 patient showed no response to treatment. Compared to a cohort of patients treated with focal radiotherapy, CSI patients were more likely to have multifocal disease at time of diagnosis (*p* = 0.001) and intradural tumor location (*p* < 0.001). Despite over-representation of these unfavorable characteristics, there was no survival difference between groups (*p* = 0.58). While CSI shows promising results in the treatment of primary intraspinal EwS, treatment should be considered individually based on tumor and patient characteristics in the absence of prospective trials.

## 1. Introduction

Skeletal tissue is the most frequent site of origin for Ewing sarcoma (EwS) in children and adolescents [1,2]. Primary extraosseous intraspinal EwS is a rare and aggressive but potentially curable malignancy [3,4]. It is classified based on its location in the spinal canal in relation to the dura mater and spinal cord as epi- or intradural and extra- or intramedullary. Intradural EwS are characterized by a high propensity for developing skip metastases.

There are no standard treatment guidelines for intraspinal EwS due to the disease’s rarity. A multimodal therapeutic approach such as (sub-)total resection with postoperative local radiotherapy (RT) and chemotherapy (CTx) seems to prolong survival in patients with primary instraspinal EwS [5,6,7,8,9,10,11]. However, the impact of neuroaxis irradiation (cranio-spinal (CSI) or spinal irradiation (SI)) on local control and survival of patients with EwS in this location remains unclear. Published data are mostly limited to case reports or case series reporting patients with primary intraspinal EwS treated with local RT to macroscopic tumor manifestations [5,6,7,8,9,10,11,12,13,14,15]. 

This study evaluates the clinical and survival outcome of patients with intraspinal EwS who received CSI. Several prognostic parameters for clinical response and OS were investigated. Survival outcome was comparatively analyzed between patients treated either with CSI or focal spinal RT. Cases with intraspinal peripheral primitive neuroectodermal tumors (pPNET) were also selected for this analysis given that pPNETs have been re-classified as EwS tumors [16].

## 2. Materials and Methods

### 2.1. Patient Population

**Figure 1:**
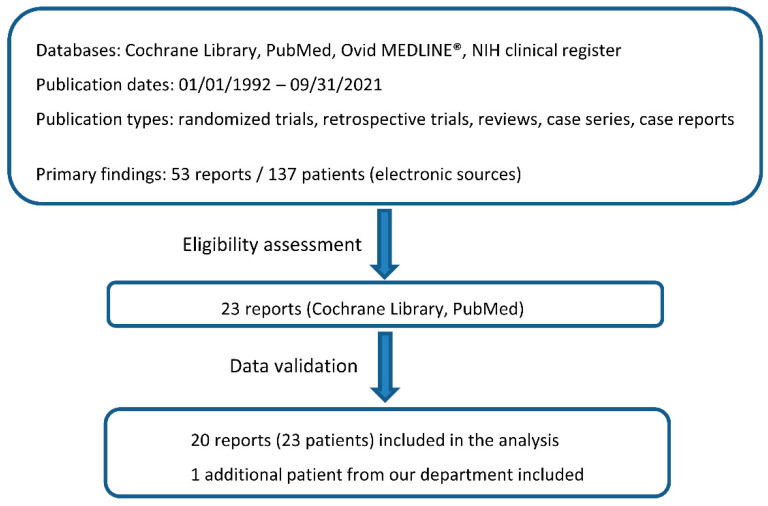
Flow chart of the report selection process. The database resources included: Cochrane Library, PubMed, Ovid MEDLINE Research platforms, and the NIH clinical trials register (https://clinicaltrials.gov/) (accessed on 4 October 2021). The following search terms were used: Ewing Sarcoma, peripheral neuroectodermal tumor, thoracal spine, lumbar spine, intraspinal Ewing sarcoma, cranio-spinal irradiation.

Given the rarity of the disease, we first performed a systematic literature review to identify a population large enough for subsequent analysis. This systematic literature review was structured according to the “PRISMA” reporting guidelines [17]. The “Seven-Step Model” was used to perform the literature search, as described in detail by Onwuegbuzie and Friels and Williams [18,19]. The corresponding flow chart is presented in Figure 1. Patients with primary extraosseous epi- and intradural EwS and pPNET which were treated with CSI as part of primary therapy were analyzed. Published trials were identified using the Cochrane Library, PubMed^®^ database and Ovid MEDLINE^®^. In addition, we searched the NIH clinical trials register (https://clinicaltrials.gov/) (accessed on 4 October 2021) to select appropriate clinical trials. The following phrases for eligibility criteria were used: “articles must be peer reviewed” and “articles must be less than thirty years old” (as of 2021). Only articles published in English were considered. For the purpose of our study, we only included reports if the radiation doses for treatment were reported. When searching for articles, we directly specified all terms used—namely, Ewing Sarcoma, peripheral neuroectodermal tumor, thoracal spine, lumbar spine, intraspinal Ewing sarcoma, cranio-spinal irradiation. 

In addition, we selected the five largest literature reviews [8,12,13,14,15] and abstracted all reports on intraspinal EwS treated with focal RT published in the last thirty years as a second group (“focal radiation group”). In accordance with our criteria for CSI patients, we only included reports that specified the local radiation dose. The same patient, tumor and treatment characteristics were collected for both groups to facilitate comparisons.

### 2.2. Data Extraction and Study Quality Assessment

Literature search, selection of studies, and data extraction were separately performed by two trained and certified radiation oncologists (S.S., K.K.). A senior physician (H.T.E.) validated the results and resolved any discrepancies concerning data assessment. 

The following patient information was extracted from the databases, if available: age, sex, date of first diagnosis, primary tumor site, date of relapse, date of death, time of irradiation, fraction and cumulative radiation dose for CSI, additional RT boost, time and mode of Cth, if/when surgical treatment, and acute and late radiogenic toxicities. Local control based on clinical and radiological response and overall survival was also collected.

### 2.3. Statistical Analysis

CSI patients are described individually in Table 1. In Table 2, data from CSI patients and 55 patients receiving focal irradiation is summarized using mean and standard deviation and median and range, as appropriate. Groups were compared for different patient, tumor and treatment characteristics via chi square test or Mann–Whitney U test, as appropriate. For survival analyses, Cox proportional hazard regression analyses were used for continuous variables while log-rank tests and Kaplan–Meier plots were performed in case of dichotomous variables. Statistical analyses were carried out with SPSS (SPSS for Windows, Version 24.0; IBM, Armonk, NY, USA) and the Stata software package (version 13.0; StataCorp, College Station, TX, USA). A *p*-value < 0.05 was considered statistically significant.

## 3. Results

### 3.1. Literature Search

Fifty-three reports were initially screened and 23 were then assessed for eligibility. A total number of 24 patients, including one patient treated in our department, were included in the analysis (Figure 1, Table 1). All reports were retrospective studies of one or multiple patients with intraspinal EwS or pPNET treated with CSI. The earliest article was published by Papadatos et al. in 1998 and the latest by Huguenard et al. in 2021 [14,20]. The studies were reported both by radiation oncology and pediatrics departments. No appropriate clinical trials in the NIH clinical trials register met inclusion criteria. 

### 3.2. Patients

Patient characteristics are summarized in Table 1. Twenty-three patients plus our own case met inclusion criteria. Twelve patients were female (50%). Median age at time of diagnosis was 25.5 years (range 2–52 years). Eight patients (33.3%) had multiple intraspinal EwS manifestations or diffuse leptomeningeal spread. The tumor was located in the intradural space in 21 cases (87.5%). Median follow-up from diagnosis was 17.5 months (range 0–120).

### 3.3. Treatment Modalities

Therapy modalities for each patient are presented in Table 1. In 20 patients (83.3%) CSI was applied after either subtotal (17 patients/70.8%) or gross intraspinal tumor resection (3 patients/12.5%). A dose escalation (boost) on the primary tumor manifestation or metastatic tumor rest was performed in 20 patients (83.3%), while the remaining 4 patients did not receive a boost. Cumulative RT on the neuroaxis ranged from 30 to 50.4 Gy (median dose 36 Gy) while the daily dose per fraction was 1.5 to 2 Gy (median dose 1.8 Gy). The radiation dose for the boost ranged from 7.2 to 29.4 Gy (median dose 14.4 Gy) with fractionation dose variations between 1.6 and 2.5 Gy (median dose 1.8 Gy). 

In 20 patients (83.3%) Cth was administrated concurrently and/or sequentially to CSI (Table 1). Most commonly, vinca-alkaloids (vincristine) and platinum-based drugs (cisplatin or carboplatin) were used simultaneously to CSI. Different combinations of vinca-alkaloids, platinum-based drugs, epipodophyllotoxins (etoposide), camptothecins (topotecan, irinotecan), anthracyclines (doxorubicin, epirubicin), antimetabolites (methotrexate), and alkylating agents (cyclophosphamide, ifosfamide, temozolomide) were given as consolidation treatment after CSI. The use of high-dose Cth (HD-Cth) with subsequent autologous stem cell transplantation was performed in 3 (12.5%) patients. The time intervals between CSI and HD-Cth as well as therapy tolerance were not described in these reports.

### 3.4. Analysis of Efficacy

Sixteen patients (66.7%) demonstrated complete radiological remission after multimodal therapy including CSI (Table 1). None of these patients died during a median follow-up of 24.5 months (range 3–120). Five patients (20.8%) showed a partial radiological remission. Three of these patients died. The median follow-up in this group was 11 months (range 0–17). Only 1 patient (4.2%) did not respond to primary therapy and was diagnosed with disease progression, dying within a year of follow-up. Time to local or distant relapse after therapy completion, therapy-related adverse effects or therapy-related death were not documented in most of the analyzed reports. One patient was documented to have developed CNS metastases after CSI.

**Table 1:** Overview of cases from the literature of primary extraskeletal intraspinal EwS treated with craniospinal RT.

Author	Sex/ Age at Diagnosis	Localization	Multifocal Tumor/ Leptomeningeal Spread	Radiotherapy		Surgery	Chemotherapy	Clinical Response	Radiological Response	Survival/Months
				CSI Cumulative/ Fraction Dose (Gy)	Boost * Cumulative/ Fraction Dose (Gy)					
Own case	F/27	intradural extramedullary	No/No	36/1.5	18/1.8	n.p.	MTX/ VCR_IFO_ETO/ HD-TREO_MEL	CR	CR	38/alive
Huguenard et al., 2021 [14]	M/34	intradural extramedullary	Yes/Yes	30.6/1.8	12.6/2.5	STR spinal lesion	CPM_CyT_VCR/ HD-IFO/ETP	PD	PD	6
Weil et al., 2001 [21]	M/21	intramedullary intracranially	Yes/No	37.8/1.8	7.2/1.8	STR cranial & spinal lesion	VCR_DXR_CPM/ ETP_ IFO	CR	CR	30/alive
Tan et al., 2019 [22]	F/34	intradural extramedullary	Yes/No	36/2	n.p.	STR	n.p.	PR, then PD	PR, then PD	11
Izubuchi et al., 2020 [15]	F/35	intradural extramedullary,	Yes/Yes	45/1.8	n.p.	STR	VCR_DXR_CPM/ IFO_ETP	PR, then PD	PR, then PD	16
Bostelmann et al., 2016 [23]	M/29	extradural	No/No	36/n.a.	14.4/1.8	GTR	VCR_IFO_DXR/ ETP_TOPO_CPM	PR	CR	18/alive
Chihak et al., 2016 [24]	M/25	intradural extramedullary	No/No	39.6/n.a.	14.4/n.a.	STR	IFO_ETP_VCR/ DXR_CPM	CR	CR	20/alive
Chihak et al. 2016 [24]	M/34	intradural extramedullary	Yes/No	30/n.a.	29.4/n.a.	STR	VCR_DXR_CPM/ IFO_ETP	CR	CR	3/alive
Khwaja et al. 2019 [25]	F/44	intramedullary	No/No	30.6/1.8	12.6/1.8	STR	CIS_CCNU_IFO/ CPM_ETP	CR	CR	96/alive
Isotalo et al., 2000 [26]	M/52	intradural extramedullary	No/No	38.5/1.75	17.5/1.75	STR	n.a.	PR	CR	12/alive
Johnson et al., 2020 [27]	M/42	intradural extramedullary,	No/Yes	37.8/1.8	16.2/1.8	STR	VCR_DXR_CPM/IFO_VCR/ TMZ_IRT	PR	PR	1/alive
Lu et al., 2019 [12]	M/25	intradural extramedullary	No/No	36/1.8	14.4/1.8	STR	IFO_VCR/ CPM_CyT	CR	CR	62/alive
Benesch et al., 2011 [28]	F/14	intramedullary	No/No	40.5/1.5	14.4/1.8	STR	CPM_VCR_ETO_CPM HD-MTX/ intrathecal MTX	CR	CR	44/alive
Benesch et al., 2011 [28]	M/2	intramedullary	Yes/Yes	35.2/1.6	9.6/1.6	STR	CAR_ETP/ CIS_ETP_VCR/ HD-MTX	PR	CR	40/alive
Takahashi et al., 2017 [29]	F/50	intramedullary	No/No	39.6/1.8	14.4/1.8	STR	CAR_ETO	PR	PR	n/a
Albrecht et al., 2003 [30]	F/29	intramedullary	Yes/No	35.2/1.6	18/1.8	n.p.	ADR_ETO_CPM	PR	PR	17
Yavuz et al., 2002 [31]	F/18	intradural extramedullary	No/No	34/n.a.	20/n.a.	STR	VCR_CPM_DXR_IFO_ ETO	CR	CR	25/alive
Izycka–Swieszewska et al., 2001 [32]	F/13	epidural	Yes/No	33/n.a.	n.p.	n.p.	CAR_EPI_VPS_VCR_IFO_ACT/ TRO_IDA_VPS	CR	CR	18/alive
Kim et al., 2004 [33]	M/17	intramedullary	No/No	50.4/1.8	n.p.	STR	n.p.	n.a.	n.a.	4/alive
Nutman et al., 2007 [34]	F/19	intradural extramedullary	No/No	36/1.5	9/1.8	GTR	CPM_VCR/ CAR_THI_ETO	CR	CR	24/alive
Papadatos et al., 1998 [20]	F/23	intradural extramedullary	No/No	36/1.5	9/1.8	STR	CPM_CIS_ETO	PR	CR	12/alive
Weber et al., 2004 [35]	M/26	epidural	No/No	36/1.8	21/1.6	GTR	VCR_ACT_CPM	CR	CR	9/alive
Gollard et al., 2011 [36]	F/21	intramedullary	No/No	36/n.a.	18/n.a.	n.p.	VCR_CIS_CPM	CR	CR	120/alive
Alexander et al., 2010 [37]	M/45	intradural extramedullary	No/No	36/1.8	18/1.8	STR	n.p.	PR	n.a.	13/alive

Abbreviations: EwS—Ewing sarcoma; PNET—peripheral primitive neuroectodermal tumor; CSI—cranio-spinal irradiation; STR—subtotal tumor resection; GTR—gross total tumor resection; HD-Ctx—high-dose chemotherapy; n.a.—detailed information is not available from the report; n.p.—not performed; CR—complete remission; PR—partial remission; PD—progressive disease; SD—stable disease; TREO—treosulfan; MEL—melphalan; ETO—etoposide; CPM—cyclophosphamide; VCR—vincristine; IFO—ifosfamide; DXR—doxorubicin; MTX—methotrexate; CIS—cisplatin; CAR—carboplatin; TMZ—temozolomide; CCNU—lomustine; IRT—irinotecan; CyT—cytoxan; ADR—adriamycin; EPI—epirubicin; VPS—vepesid; ACT—actinomycin D; TRO—trofosfamide; IDA—idarubicin; THI—thiotepa; *—Dose escalation (boost) on the primary spinal tumor manifestation.

### 3.5. Comparison with Focal Irradiation

To compare therapy efficacy of CSI and focal spinal RT in patients with intraspinal EwS we selected five large reviews [8,12,13,14,15] and identified 55 patients with EwS who received focal spinal RT with reported radiation treatment doses. References for all reports included can be found in Supplementary Document S1. Patient characteristics are summarized in Table 2 and compared to the CSI group. There were no significant differences in patient characteristics. CSI patients tended to be slightly older compared to focal irradiation patients (*p* = 0.09). However, tumor characteristics differed strongly: Ewing sarcomas treated with CSI were more likely to be located within the dura (*p* < 0.001) and were more likely to be multifocal at time of diagnosis (*p* = 0.001). More than half of CSI tumors involved multiple sections of the spine (cervical, thoracic, lumbosacral) while this was only true for one in three tumors treated with focal radiotherapy. Local radiation dose to the tumor was not significantly different between groups with a slightly higher dose in CSI group (51.8 vs. 50.0 Gy, *p* = 0.08). Conversely, dose per fraction was smaller in the CSI group (1.8 vs. 2 Gy, *p* < 0.001).

**Figure 2:**
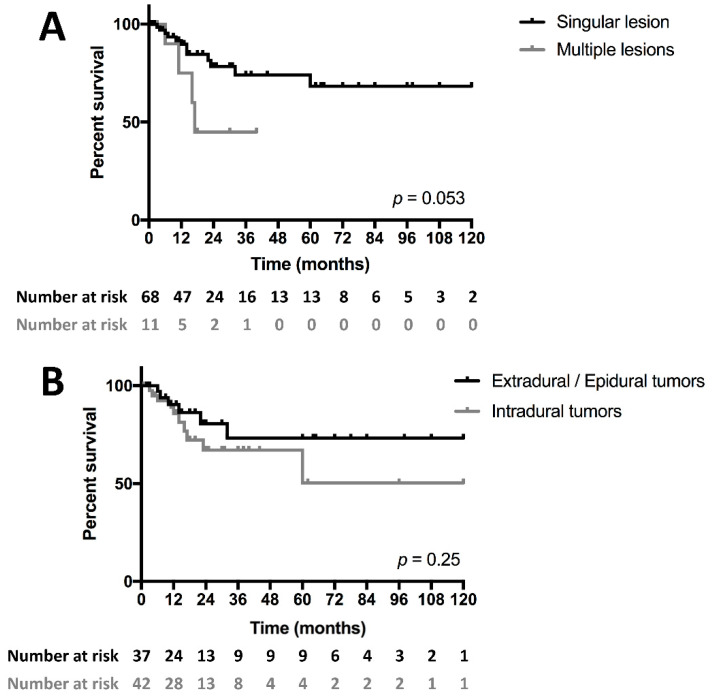
Kaplan–Meier survival curves of EwS patients with multiple intraspinal lesions at time of diagnosis compared to singular tumors (**A**) and with intradural compared with extradural/epidural tumors (**B**). Patients with multiple lesions tended to do substantially worse compared to those with singular tumors (*p* = 0.052). Intradural tumors were more likely to be associated with a worse prognosis (*p* = 0.25).

We subsequently aimed to understand associations with survival regarding patient and treatment characteristics in a combined cohort of 79 patients. We found survival among patients with multiple lesions at time of diagnosis to be substantially worse compared to those with singular tumors (*p* = 0.053, Figure 2A). Intradural tumors also tended to be associated with slightly worse outcomes compared to extradural lesions (*p* = 0.25, Figure 2B).

**Figure 3:**
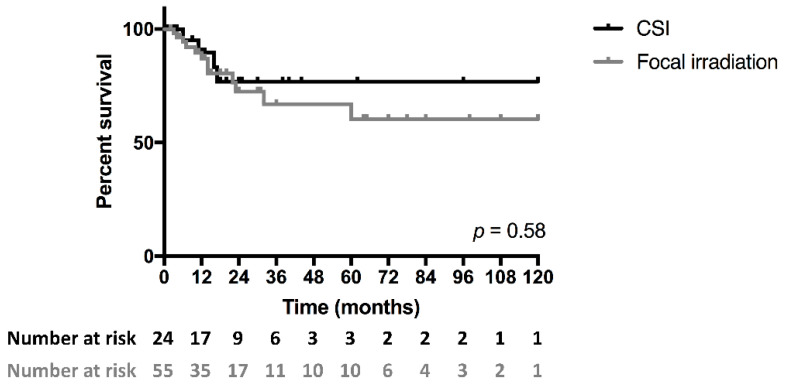
Kaplan–Meier survival curves of patients with intraspinal EwS treated either with CSI or focal RT. No significant difference between cohorts was observed (*p* = 0.58).

There was no significant different between CSI and focal irradiation groups (*p* = 0.58, Figure 3).

Finally, toxicities were not reported in most case reports, precluding detailed analyses.

**Table 2:** Patient characteristics of the focal radiotherapy group, compared to the craniospinal irradiation (CSI) group. Mean (standard deviation), median (range) or *n* (%) is displayed, as appropriate. Not all information was available for all patients.

Characteristic	Focal Radiotherapy 55 Patients	Craniospinal Irradiation 24 Patients	*p*
Age, years, median (range)	22 (1–70)	25.5 (2–52)	0.09 *
Sex			0.33 ^#^
Male, n (%)	34 (61.8)	12 (50.0)	
Female, n (%)	21 (38.2)	12 (50.0)	
Tumor localization			**<0.001 ^#^**
Intradural, n (%)	21 (38.2)	21 (87.5)	
Epidural/extradural, n (%)	34 (61.8)	3 (12.5)	
Multiple lesions or leptomeningeal spread at time of diagnosis, n (%)	3 (5.5)	8 (33.3)	**0.001 ^#^**
Localization of the tumor(s) at time of diagnosis			0.21 ^#^
Cervical spine	10 (18.2)	4 (16.7)	
Thoracic spine	9 (16.4)	4 (16.7)	
Lumbosacral spine	18 (32.7)	3 (12.5)	
Multiple segments involved	18 (32.7)	13 (54.2)	
Follow-up, months, median (range)	13 (2–120)	17.5 (0–120)	0.72 *
Death during follow-up, n (%)	12 (21.8)	4 (16.7)	0.60 ^#^
Development of neuroaxis metastases after treatment (only patients with singular tumor at time of diagnosis considered), n (%)	10 (19.2)	1 (6.3)	0.22 ^#^
Radiation dose to the tumor region (Gy)	50.0 (30.0–65.0)	51.8 (33.0–59.4)	0.08 ^#^
Dose per fraction (Gy) ^x^	2 (1.75–3)	1.8 (1.5–2)	<0.001 *

* Mann–Whitney U test; ^#^ chi square test; ^x^ for CSI patients, CSI fractionation dose was used if different from boost fractionation dose given that most fractions were applied via CSI, not via boost. Fractionation dose was available in *n* = 18 CSI patients and in *n* = 24 focal irradiation patients. *p* values meeting level of significance are printed in bold.

### 3.6. Prognostic Parameters in the CSI Group

Additional risk factors potentially relevant for therapy efficacy were also analyzed in the CSA group. Cox proportional hazard regressions were used for continuous variables while log-rank tests were performed for dichotomous variables (Table 3). 

Analyses showed that in this subgroup multifocal tumors were unfavorable for survival. Similarly, older age tended to be associated with worse prognosis. CSI dose, boost dose and sex had no influence on outcomes. Omission of a boost, however, was associated with inferior outcomes. Finally, radiologic complete response was prognostically favorable.

### 3.7. Own Case

**Figure 4:**
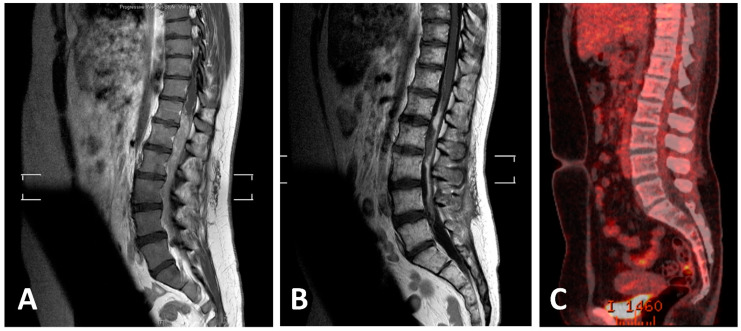
MRI and PET-CT scans of lumbar EwS before and after definitive RCth (radiochemotherapy) and ASCT (autologous stem cell transplantation). (**A**) Initial T1-weighted sagittal MRI of the thoracic and lumbar spine with intramedullary tumor; (**B**) T1-weighted sagittal MRI with residual post-therapeutic signal alterations; (**C**) post-therapeutic PET-CT scan with no residual increased FDG uptake.

One patient from our department with primary intradural extramedullary EwS disease received definitive combined radiochemotherapy (RCth) according to the European Ewing tumor Working Initiative of National Groups—Ewing Tumor Studies 1999 (EURO-E.W.I.N.G. 99) therapy protocol. The patient presented with lumbar pain, hypesthesia, and a beginning paresis of the left leg. Staging showed an intradural mass ranging from T12 to L5 (Figure 4) and malignant cells in the cerebrospinal fluid. After biopsy allowed for histopathologic diagnosis Cth with six cycles of systemic VIIE (vincristine, idarubicin, ifosfamide, etoposide) and one cycle of intrathecal methotrexate was applied. Then, definitive RT including CSI with 36 Gy in 1.5 Gy fractions and a boost to the tumor region from level T12 to L5 up to a dose of 54 Gy was performed. Consolidating chemotherapy with 4 cycles of VEI (vincristine, etoposide, and ifosfamide) and a high-dose chemotherapy (HD-Cth) was then followed by autologous stem cell transplantation. Afterwards, two additional cycles of VEI were given and FDG-PET/CT, MRI, and cerebrospinal fluid showed a complete response (Figure 4). Thirty-eight months after the end of the therapy, the latest follow-up showed no sign of new disease.

## 4. Discussion

We present the largest analysis to date to assess the value of neuroaxis irradiation in EwS patients with primary intraspinal (epi-or intradural) manifestations. Given the rarity of the disease, no prospective data exist, and most studies are limited to individual case reports. 

### 4.1. Therapeutic Regimens

Prior published case reports point towards the utility of multimodal therapy to reduce neurologic deficits as well as improve local control and progression-free survival [6,10,21,38]. Generally, most patients undergo resection first given that decompression may quickly ameliorate neurologic symptoms. However, the frequency of subtotal resection of an intraspinally located tumor is high (54% reported by Saeedinia et al. and 64% reported by Kaspers et al.) due to the rapid expansion and involvement of the meninges and medulla [8,38]. The same is true in our cohort, where 20 patients underwent surgery but only 3 complete resections were performed. Unsurprisingly, residual tumor was found to be associated with an increased rate of local and systemic recurrence and higher mortality for primary intraspinal EwS [8,38]. Despite these challenges, evidence indicates that a subtotal or at least partial resection of the tumor may be associated with improved survival, at least if coupled with postoperative chemoradiation [5,8,11,13,14,22,23,24,38,39]. 

Adjuvant chemoradiation as part of multimodal therapy is also largely recommended. Very few published cases demonstrate a good clinical response of primary epi- or intradural EwS if only treated with CTx [7,38]. Kaspers et al. achieved a complete remission for 40 months (latest examination) with no neurologic deficit exclusively with CTx without radiation treatment in a patient with partially resected primary epidural extraosseous EwS [38]. The decision to omit radiotherapy in several cases of intraspinal EwS was based on effective control through CTx and considering potential radiation-induced complications such as vertebral deformities and secondary malignancies [38,40]. However, it has been noted that the blood–brain barrier inhibits most chemotherapeutic agents to effectively access intrathecal tumors [41,42]. Thus, trimodal therapy remains the standard of care. 

Unfortunately, chemoradiation regimens remain diverse. This is underlined by the wide variation in treatments (both in choice of chemotherapy and fractionation of radiotherapy) even among our small group of patients treated with CSI. 

In summary, a substantial number of treatment uncertainties exists regarding all parts of trimodal therapy given the low incidence of the disease. In our present study, we aimed to assess the value of craniospinal radiotherapy in this setting.

### 4.2. CSI for Local and Distant Control

As noted, CSI led to a 67% radiological CR rate. It is difficult to compare this rate to focal irradiation studies due to the absence of large analyses. In our study, we see no major differences regarding radiation dose to the local tumor region between CSI and focal radiotherapy patients (50 Gy for focal radiotherapy vs. 51.8 Gy for CSI). Conversely, dose per fraction was significantly higher in the focal irradiation group, but the absolute difference was equally small (1.8 vs. 2 Gy median). Hence, biologically equivalent doses to the tumor region are similar between both groups, making it unlikely that radiation-induced local control is different between CSI and focal irradiation treatment paradigms. On the other hand, we hypothesized that failure within the spine may be a different matter given the difference in treatment fields (CSI vs. focal radiotherapy only). An analysis of 56 patients provided by Saeedinia et al. suggested a general rate of distant metastases of 32% in patients treated with surgery and adjuvant focal RT and CTh [8]. Meanwhile, risk was even higher, 46%, if no trimodal therapy was performed [8]. Thus, given high rates of metastases, reducing spinal seeding has significant clinical relevance.

While case numbers in our study are low and level of significance was not reached, patients undergoing focal irradiation were more likely to develop craniospinal metastases during follow-up than CSI patients (19% vs. 6%). The risk of a leptomeningeal spread is particularly high in intraspinal/intradural tumors [15,43]. It is promising to see the low rate of CNS metastases in the CSI group despite strong overrepresentation of intradural tumors among these patients. Based on these findings, we recommend CSI for multifocal and/or intradural EwS tumors. While it was not specifically investigated in our study, we would similarly suggest CSI to patients where tumor cells have been identified in the cerebrospinal fluid. 

### 4.3. CSI for Survival

Reports on intraspinal EwS mortality differ somewhat. Investigations have estimated a 5-year survival rate between 43% and 48% [44,45] or a mortality of 62% after 16 months [38]. A one- and two-year survival rate of 81% and 52%, respectively, was found by Saedinia et al. in the largest retrospective analysis of patients with primary intraspinal EwS by far [8]. Generally, intradural tumors presented with a worse prognosis compared to intraspinal tumors and location in the upper spine compared to the lower spine seems equally unfavorable [13].

Given the rarity of the disease, meaningful analyses of prognostically relevant patient or treatment characteristics in large cohorts are scarce except for the findings regarding the advantage of trimodal therapy discussed above [8]. Here, we found that use of CSI was not significantly different for overall survival when compared to focal irradiation. However, it is encouraging to see CSI resulting in comparable survival in log-rank analyses when compared to focal irradiation despite the stark over-representation of patients with unfavorable tumors (both multifocal and/or intradural) in the CSI group. While the data are clearly insufficient to generally suggest CSI as the preferred radiation treatment for all patients with intraspinal EwS, we believe that this radiation treatment may merit more attention in treatment discussion for this disease. Based on our literature data only a small minority of patients receive CSI at the moment.

Within the CSI group we aimed to define additional prognostically relevant characteristics. We found that young age tended to be prognostically favorable, as has previously been suggested [8]. A dose escalation to the craniospinal axis did not improve the response or OS in patients. In our cohort, we found that three patients received CSI doses of more than 40 Gy, substantially exceeding 36 Gy, the most commonly used dose. Given the high risk for side effects and the lack of data to suggest a treatment advantage for higher CSI doses, we would suggest for 36 Gy to remain the therapy standard. Similar results were observed regarding the local boost dose, where no benefit was seen for higher doses. This was in line with a previous study by Barberi et al. that showed low cumulative doses to be therapeutically sufficient [45]. Conversely, an additional boost to the primary tumor was found to be a possibly relevant parameter for overall survival compared to no boost. In practice, we suggest to routinely combine a CSI of 36 Gy with a focal boost to the tumor region.

### 4.4. Compatibility of CSI with Systemic Therapy

The large radiation field of a craniospinal irradiation limits the simultaneous application of systemic therapy. Cisplatin, carboplatin, and vincristine were most commonly applied simultaneously to CSI, but doses were reduced to account for the risk of hematotoxicity. More toxic regimens including etoposide, doxorubicin, epirubicin, methotrexate, cyclophosphamide and ifosfamide were only given sequentially to CSI. Unfortunately, timing was not commonly available in case reports. In the case of our patient, 36 Gy was applied and VEI was given sequentially without severe hematotoxic side effects. HD-Cth followed by stem cell transplantation was then performed, again without any side effects. To date, no general guidelines exist on the combination of CSI and chemotherapy. For parallel use of CSI and Cth, drugs should be selected that are less myelotoxic or have less neurologic, pulmonary, and intestinal side effects and drug doses should be reduced to account for toxicities. HD-Cth should be administered sequentially only. 

In general, neither acute nor late side effects were investigated in the case reports we analyzed. However, it is likely that CSI results in increased long-term toxicity compared to focal irradiation given the substantially expanded radiation field. The median CSI patient in our cohort was about 25 years old making it likely that even long-term side effects are relevant for patients if they survive the disease. These may include growth failure, skeletal deformities, or secondary malignancy as well as toxicities to the lung, the colon, or the central nervous system itself, e.g., cognitive decline. However, these side effects, while potentially severe, should not generally preclude use of CSI in these patients given life-limiting potential of the disease and tumor-induced neurological disorders. We believe that careful discussions with patients are necessary and advocate for use of CSI on a case-by-case basis. As discussed above, tumor characteristics should play a key role in decision-making here.

As previously noted, this study is limited by its retrospective nature and the lack of available data regarding this rare disease. Low patient numbers preclude multivariate analyses and, hence, confounding factors can be reported but not conclusively analyzed. Chief among them, patient and tumor characteristics and the multitude of different systemic and surgical treatment strategies complicate the comparison of treatment regimens. Selection biases regarding the published cases apply. 

However, in the absence of larger, potentially prospective studies, our investigation may help guide clinical decision-making regarding radiation treatment in this rare disease by compiling and analyzing scarce available data on CSI in intraspinal EwS.

Thus, further efforts to build on these findings should include (a) analyzing a larger group of patients including a long follow-up; (b) prospective evidence comparing neuroaxis irradiation followed by boost versus local RTx with intensified radiation dose; (c) improving radiotherapy techniques to protect the organs at risk (e.g., medulla, hippocampus); (d) optimizing the combination of CSI and systemic therapy; (e) investigation and comparison of intraspinal and intracranial Ewing sarcoma given potentially similar challenges.

## 5. Conclusions

This investigation is the first to focus on the use of CSI in primary intraspinal Ewing sarcoma and proposes some key findings: CSI is a feasible procedure that is often used to treat multifocal and intradural tumors. Despite overrepresentation of this prognostically unfavorable cohort in our CSI group, overall survival is comparable to focal radiotherapy. A local boost to the tumor region in addition to CSI may be prognostically favorable while there is no evidence to support CSI doses higher than 36 Gy. 

Given the absence of prospective data, no general recommendation for CSI in intraspinal Ewing sarcoma can be made. However, intradural or multifocal tumors or those with tumor cells in the cerebrospinal fluid should likely be offered CSI treatment.

## Figures and Tables

**Table 3:** Prognostic parameters in the CSI group. Cox proportional hazard regressions were used for continuous variables while log-rank tests were performed for dichotomous variables.

Characteristic	Hazard Ratio	95% Confidence Interval		*p*
Age (years)	1.07	0.98–1.17		0.11
CSI dose (Gy)	1.05	0.77–1.43		0.77
Boost dose (Gy)	1.06	0.73–1.54		0.74
**Characteristic**	***p*-Value in Log-Rank Analysis**		**Directionality**	
Sex	0.54		None	
Surgery (STR vs. GTR vs. biopsy only)	0.71		None	
Application of boost vs. no boost	**0.03**		Boost application favorable	
Radiologic response	**<0.001**		Complete response favorable vs. rest	
Localized vs. multifocal	**0.005**		Unifocal favorable vs. multifocal	

*p* values meeting level of significance are printed in bold.

## Data Availability

All data is available in the manuscript and its supplements.

## Supplementary Materials

The following supporting information can be downloaded at: https://www.mdpi.com/article/10.3390/cancers14051209/s1, Supplementary Document S1: References of reports used for the focal radiotherapy group.
