# Supplementary material for: The Role of Neuroaxis Irradiation in the Treatment of Intraspinal Ewing Sarcoma: A Review and Meta-Analysis
Source: Cancers (Basel). 2022 Feb 25;14(5):1209. doi: 10.3390/cancers14051209 (PMC8909708; doi:10.3390/cancers14051209)
Supplement: Supplementary file 1 [file cancers-14-01209-s001.zip › cancers-1560259-supplementary.pdf]

Supplementary Document 1: References of reports used for creation of the focal radiotherapy group.

1. Mottl, H.; Koutecky, J. Treatment of spinal cord tumors in children. *Med. Pediatr. Oncol.* **1997**, *29*, 293–5.

2. Kennedy, J.G.; Eustace, S.; Caulfield, R.; Fennelly, D.J.; Hurson, B.; O'Rourke, K.S. Extraskeletal Ewing's sarcoma: A case report and review of the literature. *Spine (Phila. Pa. 1976).* **2000**, *25*, 1996–1999.

3. Kadri, P.A. do S.; Mello, P.M.P. de; Olivera, J.G. de; Braga, F.M. Sarcoma de Ewing epidural lombar primário: Relato de caso. *Arq. Neuropsiquiatr.* **2002**, *60*, 145–149.

4. Albrecht, C.F.; Weiss, E.; Schulz-Schaeffer, W.J.; Albrecht, T.; Fauser, S.; Wickboldt, J.; Hess, C.F. Primary intraspinal primitive neuroectodermal tumor: report of two cases and review of the literature. *J. Neurooncol.* **2003**, *61*, 113–20.

5. Kogawa, M.; Asazuma, T.; Iso, K.; Koike, Y.; Domoto, H.; Aida, S.; Fujikawa, K. Primary cervical spinal epidural Extra-osseous Ewing's sarcoma. *Acta Neurochir. (Wien).* **2004**, *146*, 1051–1053.

6. Athanassiadou, F.; Tragiannidis, A.; Kourti, M.; Papageorgiou, T.; Kotoula, V.; Kontopoulos, V.; Christoforidis, J. Spinal epidural Extraskeletal Ewing sarcoma in an adolescent boy: A case report. *Pediatr. Hematol. Oncol.* **2006**, *23*, 263–267.

7. He, S.S.; Zhao, J.; Han, K.W.; Hou, T.S.; Nazakat, H.; Zhang, S.M. Primitive neuroectodermal tumor of lumbar spine: Case report. *Chin. Med. J. (Engl).* **2007**, *120*, 844–846.

8. Bozkurt, G.; Ayhan, S.; Turk, C.C.; Akbay, A.; Soylemezoglu, F.; Palaoglu, S. Primary extraosseous Ewing sarcoma of the cervical epidural space. Case illustration. *J. Neurosurg. Spine* **2007**, *6*, 192.

9. Erkutlu, I.; Buyukhatipoglu, H.; Alptekin, M.; Ozsarac, C.; Buyukbese, I.; Gok, A. Primary spinal epidural extraosseous Ewing's sarcoma mimicking a spinal abscess. *Pediatr. Hematol. Oncol.* **2007**, *24*, 537–542.

10. Kiatsoontorn, K.; Takami, T.; Ichinose, T.; Chokyu, I.; Tsuyuguchi, N.; Ohsawa, M.; Ohata, K. Primary epidural peripheral primitive neuroectodermal tumor of the thoracic spine - Case report. *Neurol. Med. Chir. (Tokyo).* **2009**, *49*, 542–545.

11. Dogan, S.; Leković, G.P.; Theodore, N.; Horn, E.M.; Eschbacher, J.; Rekate, H.L. Primary thoracolumbar Ewing's sarcoma presenting as isolated epidural mass. *Spine J.* **2009**, *9*, 9–14.

12. Harimaya, K.; Oda, Y.; Matsuda, S.; Tanaka, K.; Chuman, H.; Iwamoto, Y. Primitive neuroectodermal tumor and extraskeletal Ewing sarcoma arising primarily around the spinal column: report of four cases and a review of the literature. *Spine (Phila. Pa. 1976).* **2003**, *28*, 408–412.

13. Mobley, B.C.; Roulston, D.; Shah, G. V.; Bijwaard, K.E.; McKeever, P.E. Peripheral primitive neuroectodermal tumor/Ewing's sarcoma of the craniospinal vault: case reports and review. *Hum. Pathol.* **2006**, *37*, 845–853.

14. Haresh, K.P.; Chinikkatti, S.K.; Prabhakar, R.; Rishi, A.; Rath, G.K.; Sharma, D.N.; Julka, P.K. A rare case of intradural extramedullary Ewing's sarcoma with skip metastasis in the spine. *Spinal Cord* **2008**, *46*, 582–584.

15. Kim, S.W.; Shin, H. Primary intradural extraosseous Ewing's sarcoma. *J. Korean Neurosurg. Soc.* **2009**, *45*, 179–181.

16. Klimo, P.; Codd, P.J.; Grier, H.; Goumnerova, L.C. Primary pediatric intraspinal sarcomas. *J. Neurosurg. Pediatr.* **2009**, *4*, 222–229.

17. Vincentelli, F.; Caruso, G.; Figarella-Branger, D. Primary intradural Ewing's sarcoma of the cauda equina presenting with acute bleeding. *Acta Neurochir. (Wien).* **2010**, *152*, 563–564.

18. Pancucci, G.; Simal-Julian, J.A.; Plaza-Ramirez, E.; García-Marcos, R.; Mayordomo-Aranda, E.; Botella-Asunción, C. Primary extraosseous intradural spinal Ewing's sarcoma: Report of two cases. *Acta Neurochir. (Wien).* **2013**, *155*, 1229–1234.

19. Lozupone, E.; Martucci, M.; Rigante, L.; Gaudino, S.; Di Lella, G.M.; Colosimo, C. Magnetic resonance image findings of primary intradural Ewing sarcoma of the cauda equina: Case report and review of the literature. *Spine J.* **2014**, *14*, e7–e11.

20. Zhao, M.; Zhang, B.; Liang, F.; Zhang, J. Primary spinal intradural extraskeletal Ewing sarcoma mimicking a giant nerve sheath tumor: Case report and review of the literature. *Int. J. Clin. Exp. Pathol.* **2014**, *7*, 9081–9085.

21. Gong, H.S.; Huang, Q.S.; Liu, G.J.; Chen, F.H.; Zhao, H.B. Cervical primary Ewing's Sarcoma in intradural and extramedullary location and skip metastasis to cauda equina. *Turk. Neurosurg.* **2015**, *25*, 943–947.

22. Scantland, J.T.; Gondim, M.J.; Koivuniemi, A.S.; Fulkerson, D.H.; Shih, C.S. Primary Spinal Intradural Extraosseous Ewing Sarcoma in a Pediatric Patient: Case Report and Review of the Literature. *Pediatr. Neurosurg.* **2018**, *53*, 222–228.

23. Akyüz, M.; Demiral, A.N.; Gürer, I.E.; Uçar, T.; Tuncer, R.; Redfern, R. Primary primitive neuro-ectodermal tumor of cauda equina with intracranial seeding. *Acta Neurochir. (Wien).* **2004**, *146*, 525–528.

24. Bazzocchi, A.; Bacci, A.; Serchi, E.; Salerno, A.; Salizzoni, E.; Leonardi, M. Intradural extramedullary Ewing's sarcoma: Recurrence with acute clinical presentation and literature review. *Neuroradiol. J.* **2013**, *26*, 476–481.

25. Chihak, M.A.; Ahmed, S.K.; Lachance, D.H.; Nageswara Rao, A.A.; Laack, N.N. Patterns of failure and optimal radiotherapy target volumes in primary intradural extramedullary Ewing sarcoma. *Acta Oncol.* **2016**, *55*, 1057–61.

26. Hsieh, C.T.; Chiang, Y.H.; Tsai, W.C.; Sheu, L.F.; Liu, M.Y. Primary spinal epidural Ewing sarcoma: A case report and review of the literature. *Turk. J. Pediatr.* **2008**, *50*, 282–286.

27. Mukhopadhyay, P.; Gairola, M.; Sharma, M.C.; Thulkar, S.; Julka, P.K.; Rath, G.K. Primary spinal epidural extraosseous Ewing's sarcoma: Report of five cases and literature review. *Australas. Radiol.* **2001**, *45*, 372–379.

28. Sharafuddin, M.J.; Haddad, F.S.; Hitchon, P.W.; Haddad, S.F.; El-Khoury, G.Y. Treatment options in primary Ewing's sarcoma of the spine: report of seven cases and review of the literature. *Neurosurgery* **1992**, *30*, 610–8; discussion 618-9.

29. Jamjoom, A.; Naim-Ur-Rahman; Hafeez, M.A.; Jamjoom, Z.A. Primary ewing sarcoma of the spine: Report of two cases. *Ann. Saudi Med.* **1993**, *13*, 563–566.

30. Tasdemiroglu, E.; Bagatur, E.; Ayan, I.; Darendeliler, E.; Patchell, R.A. Primary spinal column

sarcomas. *Acta Neurochir. (Wien).* **1996**, *138*, 1261–1266.

31. Rock, J.; Kole, M.; Yin, F.F.; Ryu, S.; Guttierez, J.; Rosenblum, M. Radiosurgical treatment for Ewing's sarcoma of the lumbar spine: case report. *Spine (Phila. Pa. 1976).* **2002**, *27*, 471–475.

32. Duan, X.H.; Ban, X.H.; Liu, B.; Zhong, X.M.; Guo, R.M.; Zhang, F.; Liang, B.L.; Shen, J. Intraspinal primitive neuroectodermal tumor: Imaging findings in six cases. *Eur. J. Radiol.* **2011**, *80*, 426–431.

33. Fabre, E.; Guillevin, R.; Chretien, F.; Le Guerinel, C.; Duffau, H. Peripheral primitive neuroectodermal tumor of the cauda equina in an elderly patient: Case report. *J. Neurosurg. Spine* **2006**, *5*, 68–71.

34. Dorfmüller, G.; Würtz, F.G.; Umschaden, H.W.; Kleinert, R.; Ambros, P.F. Intraspinal primitive neuroectodermal tumour: report of two cases and review of the literature. *Acta Neurochir. (Wien).* **1999**, *141*, 1169–75.

35. Mawrin, C.; Synowitz, H.J.; Kirches, E.; Kutz, E.; Dietzmann, K.; Weis, S. Primary primitive neuroectodermal tumor of the spinal cord: case report and review of the literature. *Clin. Neurol. Neurosurg.* **2002**, *104*, 36–40.

36. Bohn Sarmiento, U.; Aguiar Bujanda, D.; Camacho Galán, R.; Rivero Vera, J.C.; Aguiar Morales, J. Lumbar region intra-spinal primitive neuroectodermal tumour (PNET) combined with neurofibromatosis type 1. *Clin. Transl. Oncol.* **2005**, *7*, 464–467.

37. Perry, R.; Gonzales, I.; Finlay, J.; Zacharoulis, S. Primary peripheral primitive neuroectodermal tumors of the spinal cord: Report of two cases and review of the literature. *J. Neurooncol.* **2007**, *81*, 259–264.

38. Akai, T.; Iizuka, H.; Kadoya, S.; Nojima, T.; Kohno, M. Primitive neuroectodermal tumor in the spinal epidural space - Case report. *Neurol. Med. Chir. (Tokyo).* **1998**, *38*, 508–511.
